# Triaxial detection of the neuromagnetic field using optically-pumped magnetometry: feasibility and application in children

**DOI:** 10.1016/j.neuroimage.2022.119027

**Published:** 2022-02-22

**Authors:** Elena Boto, Vishal Shah, Ryan M. Hill, Natalie Rhodes, James Osborne, Cody Doyle, Niall Holmes, Molly Rea, James Leggett, Richard Bowtell, Matthew J. Brookes

**Affiliations:** a Sir Peter Mansfield Imaging Centre, School of Physics and Astronomy, University of Nottingham, University Park, Nottingham, NG7 2RD, UK; b QuSpin Inc. 331 South 104^th^ Street, Suite 130, Louisville, Colorado, 80027, USA

## Abstract

Optically-pumped magnetometers (OPMs) are an established alternative to superconducting sensors for magnetoencephalography (MEG), offering significant advantages including flexibility to accommodate any head size, uniform coverage, free movement during scanning, better data quality and lower cost. However, OPM sensor technology remains under development; there is flexibility regarding OPM design and it is not yet clear which variant will prove most effective for MEG. Most OPM-MEG implementations have either used single-axis (equivalent to conventional MEG) or dual-axis magnetic field measurements. Here we demonstrate use of a triaxial OPM formulation, able to characterise the full 3D neuromagnetic field vector. We show that this novel sensor is able to characterise magnetic fields with high accuracy and sensitivity that matches conventional (dual-axis) OPMs. We show practicality via measurement of biomagnetic fields from both the heart and the brain. Using simulations, we demonstrate how triaxial measurement offers improved cortical coverage, especially in infants. Finally, we introduce a new 3D-printed child-friendly OPM-helmet and demonstrate feasibility of triaxial measurement in a five-year-old. In sum, the data presented demonstrate that triaxial OPMs offer a significant improvement over dual-axis variants and are likely to become the sensor of choice for future MEG systems, particularly for deployment in paediatric populations.

## 1. Introduction

Optically-pumped magnetometers (OPMs) have the potential to fundamentally change instrumentation for magnetoencephalography (MEG). Conventional MEG systems (Cohen, 1972; Hämäläinen et al., 1993) detect the neuromagnetic field using superconducting quantum interference devices (SQUIDs; Jaklevic et al., (1964)). SQUIDs have high sensitivity, but must be cryogenically cooled, which means that sensors are fixed rigidly in a one-size-fits-all helmet, and a thermally insulating gap must be maintained between the sensors and the participant. Restricted proximity to the subject lowers both signal strength (which declines with the square of distance from the brain) and spatial precision (especially in people with a small head – e.g., infants). Moreover, the fixed nature of the array not only means participants must remain still (relative to the sensors) for long periods (Gross et al., 2013), but also means inhomogeneous coverage, with sensor proximity changing with brain region. These problems place significant limitations on subject demographics (e.g., scanning children is challenging) and experimental protocols (experiments requiring significant head movement are prohibited). OPMs offer similar sensitivity to SQUIDs (Boto et al., 2017), but without the need for cryogenics. Further, newly available commercial OPMs are approximately the size and weight of a Lego-brick (Boto et al., 2019; Osborne et al., 2018). This means MEG arrays can be constructed to adapt to any head shape or size; improved proximity leads to better data with more uniform coverage and, assuming background magnetic fields are controlled to high precision (Holmes et al., 2019, 2018), sensors can move with the head during a recording (Boto et al., 2018). These significant advantages, coupled with a lower cost, make OPMs an attractive alternative to SQUIDs for MEG system design, facilitating new experimental protocols (e.g. Hill et al., (2019); Seymour et al., (2021)).

The use of OPMs for MEG measurement is now well established (Borna et al., 2020; Hill et al., 2020; Iivanainen et al., 2019; Kamada et al., 2015) and motion robustness has enabled a number of demonstrations of novel paradigms, from ball games (Boto et al., 2018) to virtual reality (Roberts et al., 2019). Flexibility of sensor placement has facilitated recordings from infants (Hill et al., 2019), and brain regions that are traditionally difficult to access (Tierney et al., 2021), whilst expanded coverage (Hill et al., 2020) enables whole-brain assessment, which is critical in applications like functional connectivity (Boto et al., 2021). Nevertheless, OPMs themselves remain nascent technology and are evolving rapidly. There are multiple ways in which sensors can be designed and fabricated, and it is not yet clear which design will ultimately prove the most beneficial for biomagnetic measurement.

At a basic level (see Tierney et al. (2019) for a review) an OPM comprises a cell containing a vapour of alkali atoms. A laser is shone through the cell and photons interact with the atoms, causing both a shift in both atomic energy level and angular momentum. In a zero-field environment and with sensors operated in the spin-exchange relaxation-free (SERF) regime, absorption of circularly polarised photons by atoms in the gas (optical pumping) causes an alignment of angular momenta, and consequently atomic magnetic moments. If zero field is maintained, the atoms become trapped in the same quantum state and their aligned magnetic moments mean that the vapour is magnetised. Once in this state, the bulk magnetisation interacts with an external field (e.g., the field from the brain) according to the Bloch Equations (Bloch, 1946). There are a number of schemes to then “read out” the local field and the scheme used in most commercial sensors relies on the fact that, around the zero-field point, the amount of light that passes through the vapour is a Lorentzian function of field (Dupont-Roc et al., 1969). This is of limited value since the symmetry of the Lorentzian means one cannot infer field direction. However, in the presence of an oscillating “modulation” field, one can measure the magnetic field component orthogonal to the laser beam (Cohen-Tannoudji et al., 1970). By using two modulation fields separated in phase or frequency, it becomes possible to measure magnetic field in two orientations, perpendicular to the beam, simultaneously.

Most OPM-MEG measurements have employed this read-out, and fields have been measured in either one orientation (usually radial to the scalp surface) or two orientations. However, recent developments have enabled an extension to this scheme. Specifically, two orthogonally-oriented laser beams can be projected simultaneously through the same cell. Judicious control of modulating fields then enables four field measurements (perpendicular to both beams). Significant challenges result from this design, since the two beams are most easily produced by a beam splitter, which halves the beam power, and thus diminishes sensitivity of the sensor. Nevertheless, if sufficient sensitivity can be achieved to measure the neuromagnetic field, such a system ostensibly allows a complete 3D vector measurement of the local magnetic field inside the vapour cell, and consequently offers the promise of a MEG system where the vector field is measured at many locations across the scalp surface.

The extent to which 3D vector field characterisation is useful for MEG is debatable. Theory (Sarvas, 1987) suggests that, assuming the head is a spherical homogeneous conductor and the extracranial space is current-free, if we know the radial component of field at all points on the spherical surface of the conductor, this can be used to derive the local magnetic scalar potential. The scalar potential can, in turn, be used to derive the 3D vector field. This means that independent measurement of tangential field components would offer no additional information, beyond what could be gained from the radial field only, and so triaxial measurement is of limited value. However, in a *practical* MEG experiment, the addition of triaxial measurement has three effects: 1) Finite spatial sampling means that we do not know the radial field everywhere, and it is possible that there could be gaps in our sensitivity to the underlying sources, particularly for shallow currents immediately beneath a radially-oriented sensor. Triaxial measurements might fill those gaps. 2) Not all magnetic fields originate from inside the brain: interference fields from other sources (either biological or environmental) will be detected and these can be better characterised (and ultimately removed) via tri-axial, rather than radial measurement (Brookes et al., 2021). 3) Triaxial sensors provide three times more measurements: tangential fields are known to be weaker than radial fields (Iivanainen et al., 2017) and so this does not equate to three times more signal; nevertheless tangential sensors can be used to boost overall signal-to-noise ratio (SNR). For these reasons, it is likely that the measurement of field vectors in MEG might offer significant practical advantages.

Triaxial measurement is not an exclusive property of OPMs. Previous work has seen construction of a complete triaxial sensor array based on SQUIDs (Haueisen et al., 2012). Furthermore, construction of a SQUID array with a small number of rotated sensors has been shown to offer practical advantages for rejection of interference (using signal space separation, Nurminen et al., (2013)). However, the construction of triaxial SQUID arrays is complicated due to the required geometry of the flux transformers. Further, each triaxial sensor requires three separately wired flux transformers, three independent SQUIDs, and associated electronics. In contrast, construction of a triaxial OPM is relatively straightforward because the three measurements can share a single cell, laser, coils and electronics. In short, triaxial OPMs can be constructed at relatively little extra cost, and so ostensibly offer an “easy” route to improving OPM-MEG efficacy by tripling the number of channels.

In this paper, we assess the viability of triaxial OPM-MEG. The paper is split into two parts: in the first part we describe the operation of a newly available, commercial (QuSpin Inc.) low-noise, triaxial OPM, and perform an evaluation of its performance – paying particular attention to its sensitivity since (as noted above) this poses a significant challenge due to the need for two orthogonal laser beams. We characterise the noise floor of the new sensor, demonstrate accuracy using phantom experiments and test sensitivity to biomagnetic fields from the heart and brain. In the second part of the paper, we assess the utility of triaxial sensors in paediatric applications. In simulation, we show why a triaxial sensor might offer better spatial coverage compared to conventional arrays. We then go on to deploy triaxial sensors to make MEG measurements in a child.

## 2. Part 1–Sensor validation

### 2.1. The triaxial sensor

**Fig. 1.**
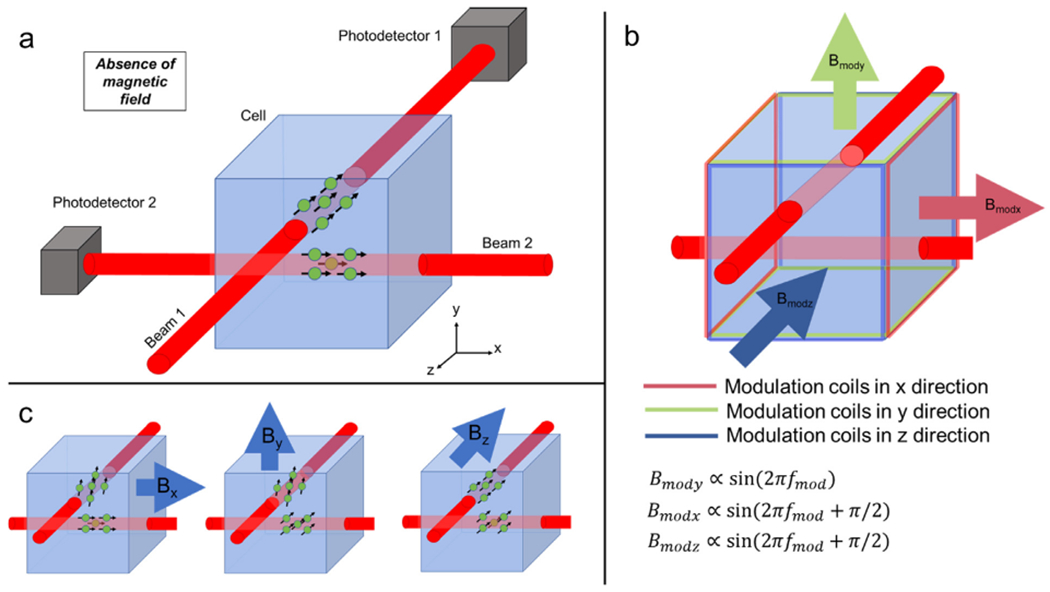
Schematic of the triaxial OPM. a) A single laser, coupled with a beam splitter, allows two independent, circularly-polarised 795 nm wavelength laser beams to be projected through a ^87^Rb cell. Both independently facilitate optical pumping. b) On-board sensor coils provide modulation fields in the x, y and z directions. A field along the axis of a beam has no effect on the atoms. Consequently, only two orthogonal modulation signals (sine waves with 0° and 90° phase shifts) are needed to read out signals in three orthogonal orientations. c) Schematic illustrations of the effect of three external (e.g. neuromagnetic) fields: the atoms in the path of Beam 1 are sensitive to fields oriented in x and y. The atoms in the path of Beam 2 are sensitive to fields oriented in y and z.

**Fig. 2.**
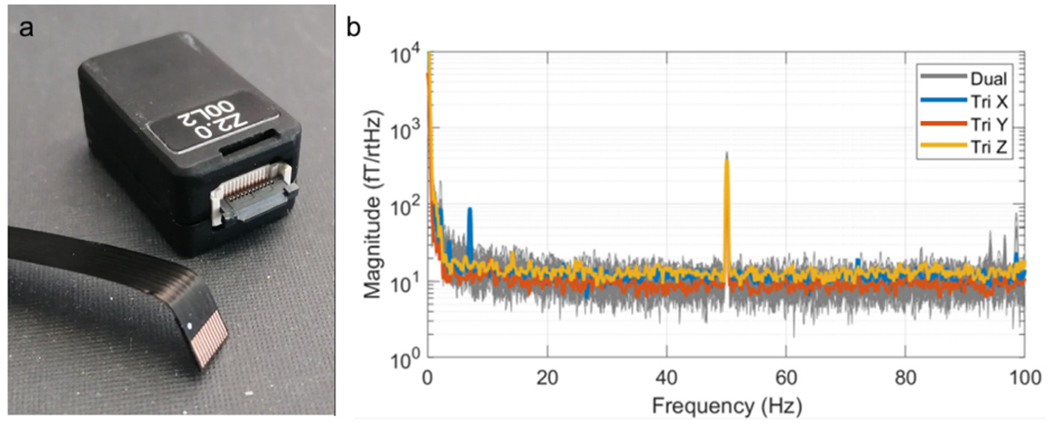
Noise spectra for the triaxial sensors. a) A QuSpin triaxial magnetometer; each sensor has a size of 1.24 cm × 1.66 cm × 2.44 cm and a weight of 7 g. Note detachable cables for ease of mounting. b) Average noise spectra for the 4 triaxial sensors (x in blue, y in orange, z in yellow) compared to dual-axis OPMs (shown in grey).

The triaxial sensor (www.quspin.com; Osborne et al., (2020)) is a single self-contained unit (Fig. 2a) comprising: 1) a glass cell (3x 3x 3 mm^3^) housing a mixture of ^87^ Rb vapour and buffer gas (nitrogen), 2) a single laser diode tuned to the D1 transition of ^87^ Rb (795-nm wavelength), with associated optics to circularly polarise the light generated, 3) two independent photodetectors and 4) three orthogonal pairs of electromagnetic coils to control magnetic field inside the cell. The laser beam is projected through a beam splitter and the two resultant beams are shone through the cell in orthogonal directions (Fig. 1a). Care is taken to ensure that the two beams have a minimal overlap (spatial separation ~0.65 ± 0.25 mm). The buffer gas minimises collisions between the Rb atoms and the cell wall, and minimises diffusion of the polarised atoms between the beams (the diffusion length over the ~3-ms coherence time (i.e., the time the atoms stay polarised) is < 1 mm). Independent photodetectors detect the amplitude of light from both beams, after passing through the cell. The two beams optically pump the Rb atoms and, in a zero-field environment, the magnetic moments of atoms align with the beam orientation (Fig. 1a). The bulk magnetisation of the atoms interacts with external magnetic fields and evolves according to the Bloch equations.

The vapour cell is enclosed in a set of three axis coils (Fig. 1b). These not only help to ensure zero field inside the cell (by nulling any residual static bias field inside the cell), but also provide oscillating modulation fields to enable lock-in amplifier-based signal processing. Take the example of Beam 1 (travelling in the *z* direction through the cell); we assume that we want to measure a field, ***B**_y_* oriented in the *y* direction, and to do so we apply a modulation field, of a form ***B**_mody_* = ***B***_1_ sin(2*πf_mod_t*), also in the *y* direction. *f_mod_* is the modulation frequency (typically ~1 kHz) and *t* represents time. The solution to the Bloch equations shows that the polarisation of atoms in the beam path is given by (Cohen-Tannoudji et al., 1970)

(1) $$P_{z}=P_{0}J_{0}\left(\frac{\gamma B_{1}}{2\pi f_{mod}}\right)J_{1}\left(\frac{\gamma B_{1}}{2\pi f_{mod}}\right)\frac{\gamma B_{y}\tau }{1+{\left(\gamma B_{y}\tau \right)}^{2}}sin(2\pi f_{{mod}^{t}}).$$

Here, ***P***_0_ is the effective equilibrium polarisation of the cell following optical pumping. *γ* is the gyromagnetic ratio and *τ* represents a time constant governing relaxation of polarisation to equilibrium. ***J***_0_ and ***J***_1_ are Bessel functions of the first kind which, in this case, collapse to constants. The signal measured at photodetector 1, ***V***_1_, is directly proportional to ***P***_*z*_, such that

(2) $$V_{1}\propto \frac{\gamma B_{y}\tau }{1+{\left(\gamma B_{y}\tau \right)}^{2}}sin(2\pi f_{{mod}^{t}})$$

A lock-in amplifier can then be used to eliminate the sinusoidal modulation (and increase SNR), leaving a signal which is proportional to $\frac{\gamma B_{y}\tau }{1+{\left(\gamma B_{y}\tau \right)}^{2}}$. Consequently, ***V***_1_ is approximately linear with field around ***B**_y_* = 0. This allows inference on the field of interest, ***B**_y_*.

In a similar way, we could measure a field oriented in the *x* direction, since symmetry suggests the solutions to the Bloch equations would be identical, as long as the modulation field is also oriented along the *x* direction. However, it is equally possible to measure the two fields (***B**_y_* and ***B**_x_*) simultaneously by applying ***B**_mody_* and ***B**_modx_* modulation fields at the same time. The two modulation fields can be applied at the same frequency and the ***B**_x_* and ***B**_y_* measurements separated by adding a 90° phase lag between *x* and *y* modulation currents. In such a scheme, we get two superimposed signals at the photodetector, the first given by Eq. (2), and the second by

(3) $$V_{2}\alpha \frac{\gamma B_{x}\tau }{1+{\left(\gamma B_{x}\tau \right)}^{2}}sin(2\pi f_{mod}+\frac{\pi }{2}).$$

The orthogonality of the sine terms, imposed by the phase offset, enables a lock-in amplifier to detect both signals independently. In this way, one can measure field at two orientations, with a single beam.

For the triaxial sensor, we have two beams which are probed by two photodetectors, independently. Beam 1 is sensitive to fields oriented in *x* and *y* (but not *z*). Beam 2 is sensitive to fields oriented in *y* and *z* (but not *x*). This is shown schematically in Fig. 1c. Note that the addition of a modulation field along the orientation of the laser beam has no first-order effect on the atoms, and so we can use the same modulation frequency and phase for the *x* and *z* directions as the atoms in Beam 1 are largely unimpacted by the *z* modulation field and the atoms in Beam 2 are similarly unaffected by the *x* modulation field. The result is two independent measures of ***B**_y_* (which are averaged together) alongside a single, simultaneous, measurement of ***B**_x_* and ***B**_z_*. Combining these provides a complete triaxial field characterisation.

### 2.2. Methods

Our aim in part one of the paper is to evaluate this form of triaxial OPM sensor, demonstrating that:

1. It offers an accurate record of magnetic field vectors
2. It has sufficient sensitivity to assess biomagnetic fields.

With these aims in mind, we undertook four experiments:

#### 2.2.1. Noise measurements

Four triaxial OPMs (QuSpin Inc. CO, USA) were operated in a single array. The sensors were placed at the centre of a magnetically-shielded room (MSR) in an ambient background field of approximately 2 nT. A separate array of 11 standard (i.e., single-beam and dual-axis) OPMs were positioned at approximately the same location. 90 s of data were simultaneously recorded from both arrays, at a sample frequency of 1,200 Hz, using a National Instruments digital acquisition system interfaced to a PC. The resulting data were segmented into 10-s windows. The power spectral density within each window was computed using the Matlab (Mathworks Inc.) ‘periodogram’ function with a flattop window, over a frequency range 0 to 100 Hz (resolution of 0.1 Hz). The median of the power spectral density across windows was then taken and plotted, providing an accurate representation of the sensor noise floor as a function of frequency. The triaxial sensors were compared to the dual-axis sensors to probe the consistency of noise floor across the two sensor types.

#### 2.2.2. Phantom measurements

**Fig. 3.**
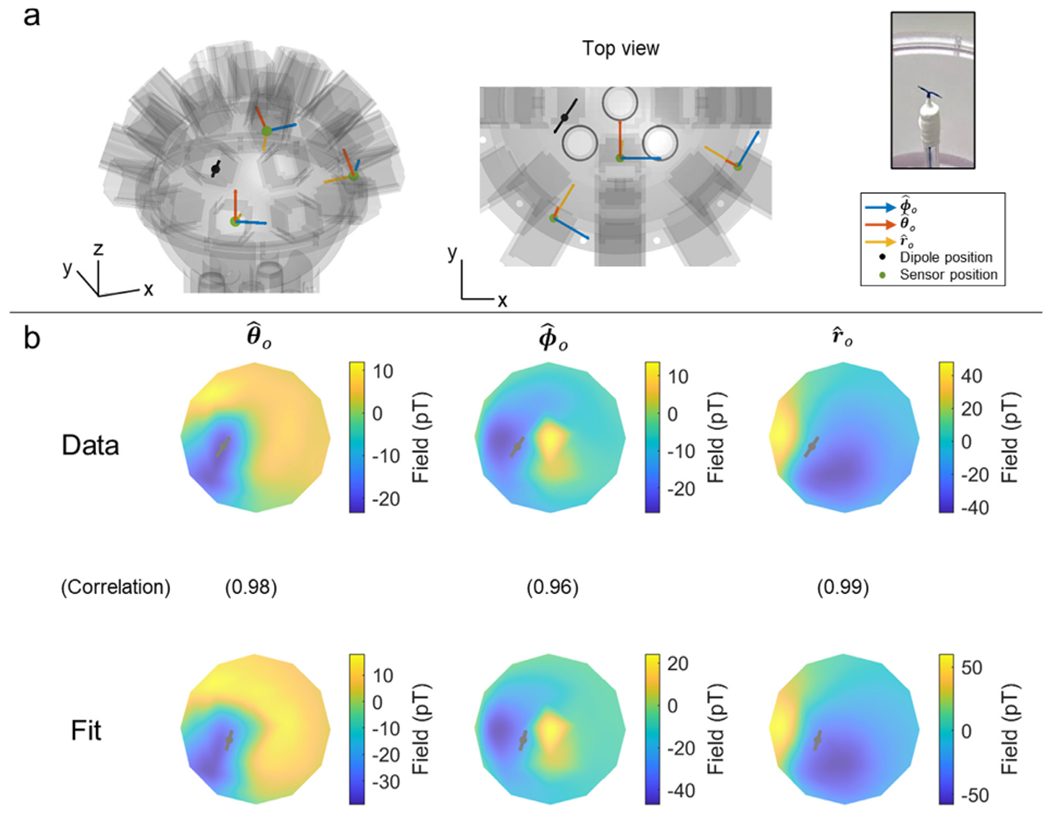
Dipole phantom experiment. a) The phantom comprised a saline filled sphere of radius 11 cm. The current dipole (black line) was formed from a twisted pair, splayed at one end (see photograph on the right). Examples of the 3 sensor orientations are shown in blue $\left({\hat{\phi }}_{0}\right)$, red $\left({\hat{\theta }}_{0}\right)$ and yellow $\left({\hat{r}}_{0}\right)$ b) 2D flattened field maps in ${\hat{\theta }}_{0}$ (left), ${\hat{\phi }}_{0}$ (middle) and ${\hat{r}}_{0}$ (right). The top row shows the measurement using triaxial OPMs, the bottom row shows the model following a dipole fit. Note the good agreement. Values in brackets represent the correlation between the two field maps (for a representative time point). On average the discrepancy between the fitted dipole and the “true” dipole location (as assessed by Polhemus digitisation) was 5.17 ± 0.04 mm.

A current dipole phantom (Fig. 3a) was used to generate a magnetic field with a known spatial signature. The phantom itself was a 3D-printed (Chalk Studios Ltd.) nylon sphere of radius 5.5 cm containing a saline solution (8 % concentration). The sphere was surrounded by 25 slots into which OPMs could be inserted. Slots were (approximately) uniformly distributed over the upper hemisphere and oriented such fields along the radial $\left({\hat{r}}_{0}\right)$, polar $\left({\hat{\theta }}_{0}\right)$ and azimuth $\left({\hat{\phi }}_{0}\right)$ orientations could be measured. (The subscript ‘*o*‘ denotes that these orientations are defined at the location of the OPM sensitive volume.) A current dipole was made from two insulated copper wires, twisted together, and separated such that the two uninsulated ends were approximately positioned 1 cm apart in the saline solution. When driven, the potential difference between the two ends of the dipole causes an ionic current to flow through the saline, which mimics a biological current dipole. The dipole was driven using a 7-Hz sinusoidal waveform produced by a signal generator, with amplitude 2 V. Currents were attenuated by a 10-kΩ resistor wired in series, to give a dipole strength of approximately 2 *μ*Am.

Four triaxial OPMs were used to measure magnetic fields from the dipole. 5 s of data were recorded at a sample rate of 1,200 Hz. This experiment was repeated 8 times with 3 of the OPMs in different positions on each repeat (1 OPM stayed in the same position for all 8 runs). Data were temporally realigned to form a single dataset, giving the impression of a single run with 25 OPMs spaced evenly around the phantom surface.

Data were processed using a bespoke dipole fitting algorithm written in Matlab. First, data were extracted at a single point in time corresponding to either a peak or trough in the sinusoidally-modulating dipole current. This resulted in 75 field measurements (i.e. 25 slots, each with three axes of measurement). A point dipole model (Sarvas, 1987) was used to simulate the expected fields. The modelled dipole was allowed to shift in location and orientation (the latter was constrained to be tangential) until the correlation between the measured data and the modelled data was maximised. This dipole fitting process was repeated for all of the peaks and troughs in the signal to get a cloud of fitted dipole locations. We noted the fitted dipole position and compared it to the “true” dipole location, which was estimated using a 3D digitiser. Specifically, the phantom was deconstructed and the location of the dipole inside mapped using a Polhemus digitiser (relative to the phantom surface and OPM locations/orientations). The locations of the cloud of points representing the dipole location were averaged to find a single location representing the centre of mass. Localisation error was taken as the Euclidean distance between the (Polhemus-derived) centre of mass and the fitted dipole location. Correlation values between the simulated field (from the fitted dipole location/orientation) and the measured fields then provided a useful quantification of the extent to which the triaxial OPM offers a true record of 3D field distribution. This process was repeated four times: using all three axes of every sensor (triaxial measurement), using just the radial and polar axes (dual axis 1), using just the radial and azimuthal axes (dual axis 2) and using radial fields only (single axis).

#### 2.2.3. Cardiac signals

**Fig. 4.**
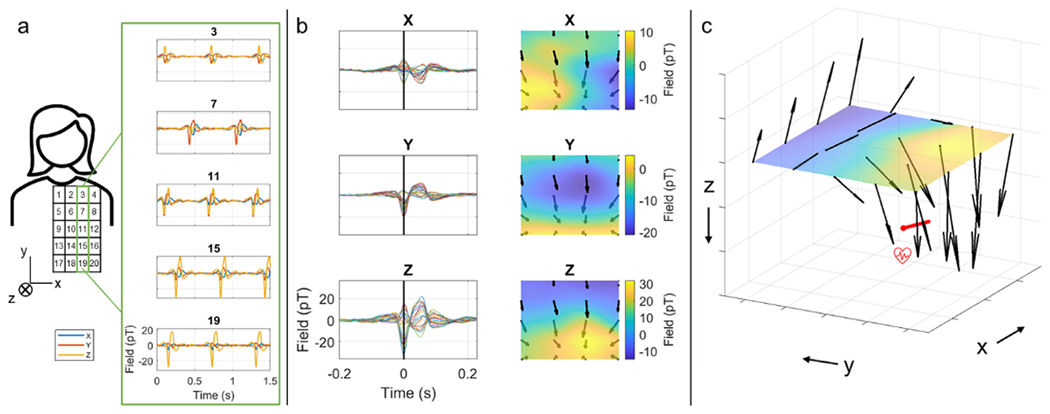
The vector field induced by the heart. a) Schematic showing the experimental set-up. 4 OPMs were placed at 20 locations above the chest (approximately over the heart). The OPMs were moved sequentially (from top to bottom) to measure the magnetic fields from the heart. Inset shows heart signal (filtered [5–45] Hz) from a single sensor at 5 different locations (*x* in blue, *y* in orange, *z* in yellow). b) Left panels show an average over cardiac cycles of the PQRST waves for x (top), y (middle) and z (bottom) magnetic field components for the 20 sensor locations. Right panels show the corresponding 2D field maps at time zero, corresponding to the time of the R-peak. c) A reconstruction of the vector magnetic fields from the heart at time zero. Note that the orientation of the fields suggests current flow along the *x*-direction in the chest as expected (represented by the red arrow).

Four triaxial OPMs were used to map the magnetic field generated by the heart. A participant stood inside the MSR, leaning against a medium-density fibreboard (MDF) sheet that allowed the OPMs to be located at 20 locations on a 4 × 5 grid (see schematic in Fig. 4a). The grid covered approximately an area of 20 cm × 20 cm. The OPMs were sequentially moved (5 times) between locations and 10 s of triaxial field data were recorded at each location. A dual-axis OPM was located on the participant’s chest and remained stationary; this was used as a reference to obtain the timing of each heartbeat. The experiment was approved by the University of Nottingham Medical School Research Ethics Committee.

The data recorded at each location were temporally realigned, using the reference OPM, such that time t = 0 was set to the QRS complex of the heartbeat. This temporal realignment allowed the formation of a dataset in which the simultaneous variation of magnetic field over a single heartbeat is shown at 20 locations (whereas in reality the measures at different locations were recorded sequentially). Triaxial fields were then reconstructed and visualised. SNR was estimated as the amplitude of the QRS complex divided by the standard deviation of the signal measured in the TP interval. All analyses were coded in Matlab.

#### 2.2.4. Triaxial neuromagnetic signals

A single participant repeated the same motor task 4 times. Upon hearing an auditory cue, the subject made two abductions of their right index finger. The inter-trial interval was set to 5 s and a single experiment comprised 50 trials. The experiment was approved by the University of Nottingham Medical School Research Ethics Committee.

**Fig. 5.**
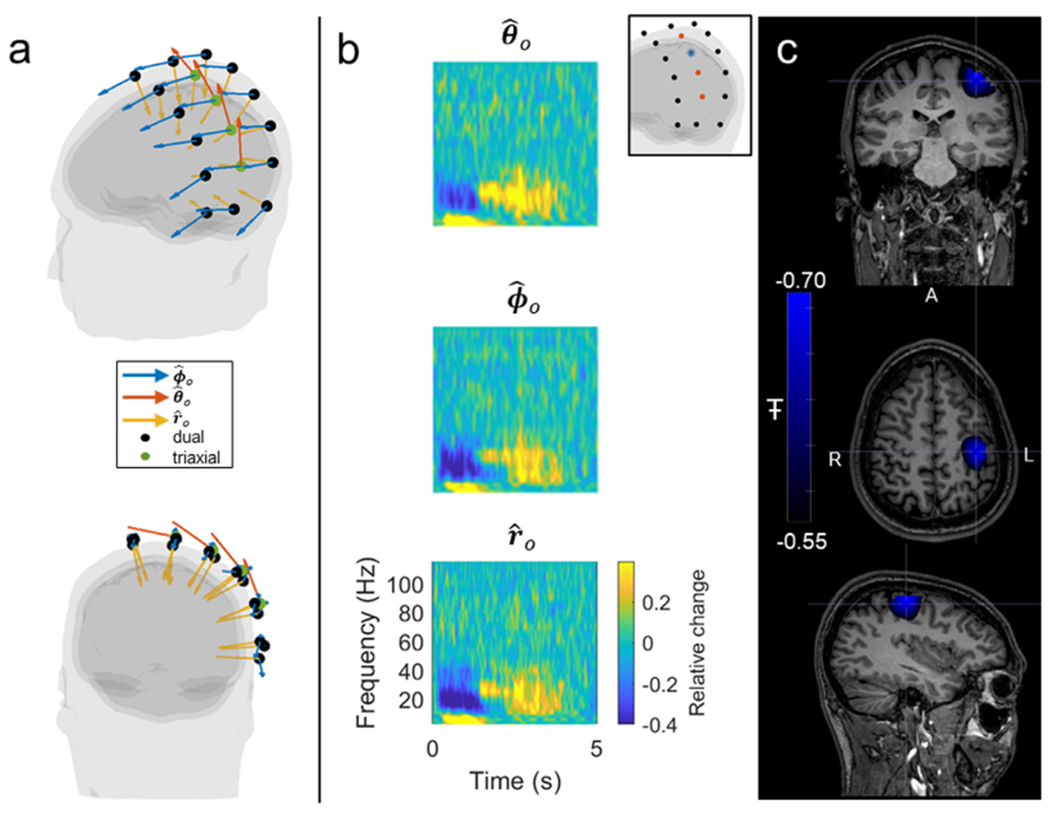
Triaxial source localisation. a) OPMs were placed in a flexible cap approximately covering the left sensorimotor cortex. Four triaxial sensors (green dots) were used in an array of 18 sensors, the remaining 14 being dual-axis sensors (black dots). b) Raw data from a single triaxial sensor (blue star inset) showing the beta response. Fields in the ${\hat{\theta }}_{0}$ (top), ${\hat{\phi }}_{0}$ (middle) and ${\hat{r}}_{0}$ (bottom) orientations are shown. c) Source images reconstructed from triaxial data using a vector beamformer approach, showing the location of maximum beta modulation in the cortex.

The OPM array (Fig. 5a) comprised 4 triaxial sensors, alongside 14 dual-axis OPMs, mounted in a flexible cap (Hill et al., 2020) at locations approximately covering the left primary sensorimotor regions and orientations (approximately) in ${\hat{r}}_{0}$, ${\hat{\theta }}_{0}$ and ${\hat{\phi }}_{0}$. The 4 triaxial sensors were placed in a row, aligned in the superior-inferior direction, approximately along the motor strip. Data were recorded from all OPMs simultaneously at a sampling frequency of 1,200 Hz. Prior to the experiment, the MSR was degaussed and a set of bi-planar electromagnetic coils used, alongside a reference array, to null the remnant magnetic field in a volume enclosing the head, to a level below ~0.5 nT (Holmes et al, 2018). The movement of the participant’s finger was recorded throughout the experiment using a 3D optical tracking system (OptiTrack, Natural Point Inc., Corvalis, US). The locations and orientations of the sensors on the participant’s head were measured using a 3D digitiser (Polhemus).

Data were initially processed in sensor space. For each channel, a time-frequency spectrum (TFS) was derived showing the evolution of oscillatory signal amplitude throughout the trial. Specifically, data were filtered into a series of overlapping frequency bands; within each band, the Hilbert transform was used to derive the analytic signal, which in turn provided an estimate of the instantaneous signal amplitude. This was averaged across trials and concatenated in frequency to yield a final trial-averaged TFS for each channel.

Following this, a vector beamformer, coded in Matlab, was applied. Initially, all OPM data (i.e., all sensors and all orientations) were frequency filtered to the 13–30 Hz band and a data covariance matrix generated. This matrix was regularised using the Tikhonov method (regularisation parameter 5 % of the maximum eigenvalue of the unregularised matrix) and beamformer weights constructed using a forward model incorporating a dipole in a single-shell conductor (Nolte, 2003), which was implemented in FieldTrip (Oostenveld et al., 2011). The vector beamformer was used to reconstruct source estimates in the polar and azimuthal orientations (the radial orientation was ignored due to the relative insensitivity of MEG to radial dipoles). A pseudo-T-statistical approach was used to construct a functional image showing the location of current dipoles exhibiting the maximum beta amplitude modulation between active (0.2 s < *t* < 1.2 s relative to movement onset) and control (2 s < *t* < 3 s) time windows. Having derived the location of maximum beta modulation, source time courses, showing the evolution of beta-band activity, were constructed in the polar $\left({\hat{\phi }}_{s}\right)$ and azimuthal $\left({\hat{\phi }}_{s}\right)$ orientations (at the peak location). Subscript ‘*s*’ indicates that these unit vectors represent orientation at the source location. Individual trial data were visualised, alongside trial-averaged data which were generated by first computing the Hilbert transform of the data to generate the analytic signal; the absolute value of the analytic signal then provided an amplitude envelope measure which was averaged across trials.

To further visualise the 3D nature of the neuromagnetic field, we undertook a “beta-burst” analysis. In recent years, the “classical” view that beta oscillations represent ongoing rhythmic activity has been challenged, by a theory suggesting that beta dynamics can be explained by the occurrence of short punctate bursts of activity whose frequency content intersects with the canonical beta range (Little et al., 2019; Seedat et al., 2020). To identify the bursts, we first calculated the amplitude envelope of the source-localised data (for the dipole in the $\hat{\phi }$ orientation) and applied a threshold at three standard deviations; this resulted in a binary time course showing periods of high beta activity Little et al., 2019. Within each identified burst, we found the time point at which the maximum amplitude of the envelope occurred. Then, a Hilbert transform was used to compute the estimated instantaneous phase of the signal and we selected the time points with a phase of zero, closest to the point of maximum amplitude. This enabled a phase alignment of the bursts. Data were then averaged across all identified bursts to derive a single representative burst time course at the dipole location. We then plotted the raw magnetic field data to visualise the magnetic field at the scalp level, produced by a dipole located in primary sensorimotor cortex and oriented azimuthally. Note we chose the $\hat{\phi }-\mathrm{oriented}$ dipole specifically, since it was orthogonal to the line joining the 4 triaxial sensors, and we expected this to give the largest field at these sensors of interest.

### 2.3. Results

The power spectral density of empty room noise is shown in Fig. 2b: traces in blue, orange and yellow show the average *x*-, *y* - and *z*-oriented measurements respectively from all four triaxial sensors. The grey traces show data from 12 standard (dual-axis) OPMs for comparison. Importantly, the noise level of an OPM depends on the strength of polarisation of atoms in the cell, which in turn depends on the power of the laser beam. Thus, one disadvantage of the triaxial approach (Fig. 1) is that splitting the laser beam halves the power, and consequently one might expect a higher noise floor in triaxial sensors compared to dual axis sensors (where the beam has higher power). However, as shown in Fig. 2b this is not the case: the noise floor for all three axes of the triaxial OPMs is comparable to that from the dual-axis array. Note that data in the *y* orientation (orange trace) has a measurably lower noise floor – this is because two independent measurements of the field in this orientation are combined (one for each beam). Quantitatively, in the [10–90] Hz range (excluding [45–55] Hz) the noise floor was measured at 13.5 ± 0.8 fT/rtHz in *x* (mean ± standard deviation over the 4 sensors) 9.9 ± 1.4 fT/rtHz in *y* and 14.9 ± 2.0 fT/rtHz in *z*. In comparison, the equivalent noise levels for the dual-axis array were 11.3 ± 1.5 fT/rtHz in *y* and 13.8 ± 1.1 fT/rtHz in *z* (mean ± standard deviation over the 14 sensors).

Fig. 3 shows results from the phantom experiment. Panel 3a shows a digital representation of the phantom with the dipole superimposed. The dipole was approximately 1 cm in length, positioned in the left hemisphere at a depth of ~ 3 cm, and oriented tangentially. On average across 68 temporally separate measurements (i.e. 34 peaks and troughs in the sinusoidal dipole signal) the dipole fit localised the dipole to within 5.17 ± 0.04 mm of the *true* location of the centre of mass of the dipole (assessed by digitisation using the Polhemus system). Fig. 3.b shows example data: the three field maps show a visual representation of field in the three orthogonal orientations (${\hat{r}}_{0}$, ${\hat{\theta }}_{0}$ and ${\hat{\phi }}_{0}$). The top row shows the measured data and the bottom row shows the modelled data. Assessment of the correlation between the measurement and model field demonstrated good agreement, with correlation coefficients of 0.9879 ± 0.0004 in ${\hat{r}}_{0}$, 0.983 ± 0.001 in ${\hat{\theta }}_{0}$ and 0.965 ± 0.001 in ${\hat{\phi }}_{0}$. Given the inherent inaccuracies in the experiment itself (see also Discussion), this level of agreement is impressive and shows that the measured fields from the triaxial array agree closely with those theoretically expected (i.e. the triaxial sensors provide an accurate representation of true field vector from the dipole).

For completeness, we also performed the dipole fit using the same data, but only 2 axis (i.e. ignoring one component of field at each sensor) or single axis (ignoring two components of field) measurement. When fitting the dipole to data in ${\hat{r}}_{0}$ and ${\hat{\theta }}_{0}$, the dipole fit error was 4.65 ± 0.05 mm, and the correlations were 0.9878 ± 0.0004 and 0.9846 ± 0.0006 in ${\hat{r}}_{0}$ and ${\hat{\theta }}_{0}$, respectively; when fitting to data in ${\hat{r}}_{0}$ and ${\hat{\phi }}_{0}$, the dipole fit error was 5.67 ± 0.05 mm and the correlations were 0.9875 ± 0.0004 and 0.967 ± 0.001 in ${\hat{r}}_{0}$ and ${\hat{\phi }}_{0}$ respectively; when fitting to data in $\hat{r}$ only, the dipole fit error was 5.11 ± 0.06 mm and the correlation was 0.9882 ± 0.0004. As expected, reducing the data to fewer field components has little effect (this will also be addressed in the Discussion).

Fig. 4 shows the results of the cardiac measurement. Panel 4a shows a schematic of the experimental set-up: triaxial OPMs were placed in 20 locations above the participant’s chest. Panel b shows a visualisation of the data recorded for a single heartbeat (PQRST waves). Here, the *z* axis runs (approximately) anterior-posterior; the *x* axis runs left-right, and the *y* axis runs (approximately) inferior-superior. The traces on the left show the measured time course of a single heartbeat whilst the field maps on the right depict the field distributions, in the *x*, *y* and *z* orientations, at the peak of the QRS complex (marked with a black line on the left traces). Panel c shows a full 3D visualisation of the measured magnetic fields, again taken from the peak of the QRS complex. The red arrow is an approximate representation of the location of the heart dipole. Importantly, we see that the triaxial sensors detect the magnetocardiogram signal with high SNR (41 for the best sensor location/orientation) as would be expected given the low noise floors demonstrated in Fig. 2b. In addition, the 3D field map shows the expected pattern, with magnetic fields rotating around the heart dipole.

**Fig. 6.**
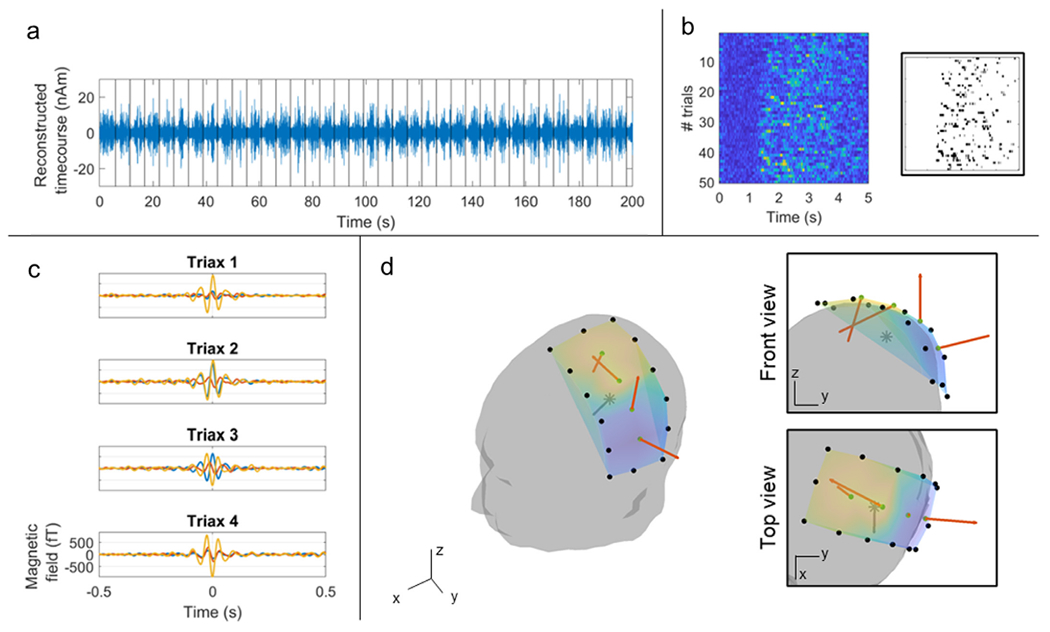
Triaxial neuromagnetic fields. a) Beamformer-reconstructed dipole time course showing the beta response in individual trials (trial onsets marked as black lines). b) Raster-plot showing the occurrence of beta bursts across trials and time. The bursts are highlighted in black in the inset plot. c) Temporal evolution of the average beta burst, measured by the four triaxial OPMs. d) Visualisation of the magnetic field vectors at the peak in beta burst amplitude (t = 0 in panel c) for the 4 triaxial sensors. The estimated dipole location (derived from our beamformer analysis) is represented with a grey star. Neuromagnetic field vectors at the four triaxial locations are marked in orange.

MEG results are shown in Figs. 5 and 6. Fig. 5a shows the digitised locations and orientations of the sensors relative to the head. Recall that four triaxial sensors (green dots) are used in an array with 14 dual-axis sensors (black dots). The orientation sensitivities of all sensors are shown by the arrows; note the four triaxial sensors are the only sensors to have sensitivity in the polar orientation, denoted by the orange arrows. The array approximately covers the participant’s left sensorimotor strip. Fig. 5b shows example TFS from a single triaxial sensor (location shown inset). In the TFS, blue denotes a decrease in oscillatory power relative to baseline, yellow denotes an increase, and time-point-zero represents the cue of movement. The data show that, in addition to cardiac signals, the triaxial sensors exhibit good sensitivity to brain activity, with the expected beta band power loss during movement followed by a post-movement “rebound” measurable across all three axes, with high SNR. Panel c shows source-space images of the spatial signature of maximum beta modulation. Again as expected, the effect localises to the primary sensorimotor regions and the result shows that the triaxial sensors can be integrated into a dual-axis array and successfully used for source localisation (obviously with only four available, we cannot perform source localisation with only triaxial sensors).

Fig. 6a shows a beamformer-reconstructed time course of beta band activity, for a modelled azimuthally-oriented $\left({\hat{\phi }}_{s}\right)$ source in left sensorimotor cortex. These data have not been averaged across trials, and the response to every trial is clearly delineated (trial onsets marked by the black vertical lines). Fig. 6.b shows the Hilbert amplitude of these same data, restructured into a raster plot with time on the *x* axis and trial number on the *y* axis. Note that, distinct from the “classical” desynchronisation and rebound which is implied by the TFS in Fig. 5b, we see that the structure of the beta modulation differs on every trial, with bursts clearly delineated. The burst structure is shown inset with a binary raster plot showing the timing of each of the beta bursts across time and trials. Fig. 6c shows the average beta burst for the azimuthal motor cortex source; the sensor-level data are shown for the 4 triaxial sensors. Finally, Fig. 6d shows a visualisation of the magnetic field, measured by each of the 4 sensors at the peak of the beta burst (time-point-zero in Fig. 6c). The location and orientation of the modelled source is also shown (in the top inset image - front view, the dipole orientation is going out of the page). Here, as with the cardiac data in Fig. 4, we see again that the magnetic field behaves as expected, rotating around the azimuthally-oriented source.

## 3. Part 2–Triaxial applications

The benefits of triaxial sensors have been explained in previous papers. At a simple level, triaxial measurements triple the sensor count (compared to radial sensors), enabling a larger number of signals over which signal-processing algorithms (e.g. source localisation) can be applied. Indeed, there is good evidence (Brookes et al., 2021; Nurminen et al., 2013) that the additional information gained by measurement of the field vector enables better differentiation of magnetic field patterns from neural sources and external (to the head) interference, thus improving rejection of signals of no interest. However, one relatively untapped area in which triaxial measurement might be important is in maximising coverage of the sensor array across the brain. Specifically, a single-axis radially-oriented sensor is insensitive to current sources directly beneath it. This is not a problem in conventional MEG because the sensors are sited a relatively large distance from the brain, and consequently the radially-oriented field is sufficiently spatially diffused that the field from a source under a sensor is readily picked up by adjacent sensors. However, as sensors get closer to the brain, the spatial frequencies characterising the field variation become higher, and the gaps between sensors can cause inhomogeneity of spatial sampling (i.e., spatial aliasing). To demonstrate this effect, and how it can be ameliorated using triaxial field sensitivity, we employed a simulation.

### 3.1. Infant simulations

#### 3.1.1. Methods

**Fig. 7.**
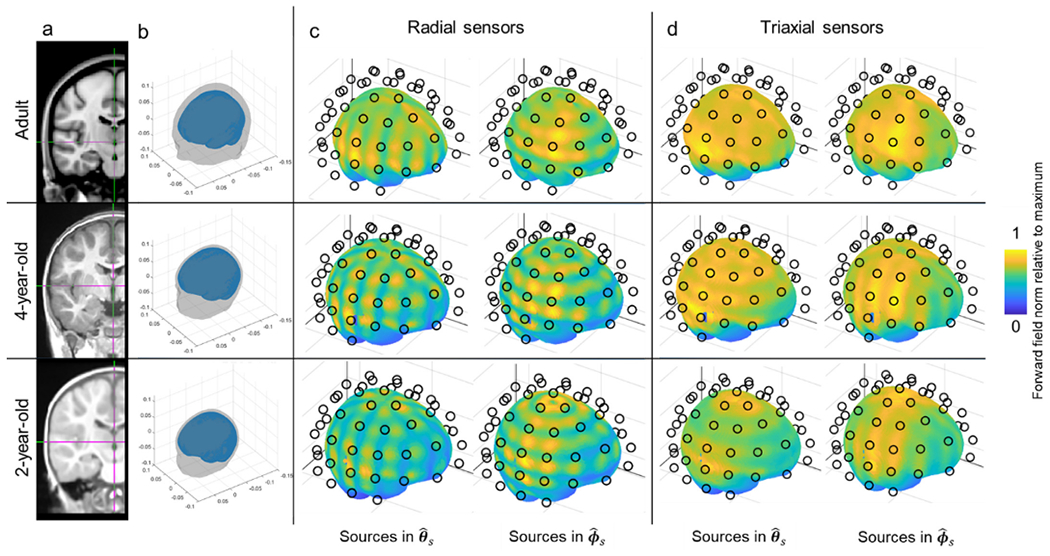
Spatial coverage simulations. a) A template MRI. b) 3D rendering of the head geometry used for the simulation. c) Array sensitivity as a function of location in the brain for a radial OPM-MEG array. Brain dipoles are positioned 5 mm beneath the brain surface (approximately in the cortex). The black circles show the locations of the OPM sensitive volumes. The colour scale represents normalised sensitivity (i.e. a value close to 1 everywhere would indicate uniform coverage; a value of 0.5 would indicate that this region only picks up 50 % of the total signal compared to the best sampled region). The left-hand column shows the case for dipoles oriented in ${\hat{\theta }}_{s}$, the right-hand column shows dipoles oriented in ${\hat{\phi }}_{s}$. d) Equivalent to c but for a triaxial sensor array. In all cases the upper, centre and lower columns show the adult, 4-year-old, and 2-year-old, respectively.

Our simulations (all implemented in Matlab) were based on three anatomical models derived from template MRIs. The templates provided an average head geometry for a 2-year-old, a 4-year-old, and an adult (Young et al., 2017). In each case, a segmentation was applied to derive a surface mesh representing the scalp and the outer brain; segmentation was performed using Fieldtrip (Oostenveld et al., 2011). Fig. 7a shows the initial MRI scan, and Fig. 7b shows a 3D rendering of the head geometry. In both cases the top panel shows the adult head, while the centre and lower panels show the 4-year-old and 2-year-old heads, respectively.

As expected, head size grows with age (approximate head circumferences are 58 cm for the adult, 50 cm for the 4-year-old, and 47 cm for the 2-year-old). However, a more dramatic change with age is the proximity of the brain to the scalp surface. The average distance from the scalp to the brain is around 15 mm in an adult, but can be as low as 5 mm (in some brain regions) in a 2-year-old (Beauchamp et al., 2011; Davis, 2021). This means that an infant/child head is not simply a scaled down version of an adult’s head, and this “non-linearity” is the origin of the MEG spatial aliasing problem.

We simulated sensor locations around the head by first fitting a sphere to the scalp and placing 77 equally-spaced points on the sphere surface. These locations were then shifted in the radial direction (relative to the sphere) to a point intersecting the scalp. This was taken as the location at which the sensor casing meets the head. The sensitive volume of the sensor (i.e., where the field measurement was actually made) was assumed to be 6 mm above the scalp, projected radially. Sensors on the underside of the sphere were eliminated to yield a realistic sampling array. 57 sensors were simulated on the adult head, 55 on the 4-year-old, and 57 on the 2-year-old. The differences were due to the way in which the algorithm equidistantly spaces points on a sphere. The sensor count of around 50 OPMs is reflective of a pragmatic OPM array which could be fabricated (Boto et al., 2021; Hill et al., 2020). Sensor orientations were defined such that fields could be measured along the radial $\left({\hat{r}}_{0}\right)$, polar $\left({\hat{\theta }}_{0}\right)$ and azimuthal $\left({\hat{\phi }}_{0}\right)$ orientations with respect to the sphere.

We aimed to investigate the extent to which this sensor array covers a set of shallow dipolar sources just beneath the brain surface. To this end, a binary volumetric image of the brain volume was extracted and eroded using a 5 mm cubic structuring element. The outer surface of the new volume was then calculated. Points on this new surface represent candidate locations for dipoles at a depth of 5 mm into the brain. Dipoles at all locations on this surface were oriented along either the polar $\left({\hat{\theta }}_{s}\right)$ or azimuthal directions $\text{(}{\hat{\phi }}_{s}\text{)}$ with respect to the best-fitting sphere. Subscript ‘*s*’ indicates orientation computed at the *source* locations. 44,803 dipole locations were simulated for the adult, 43,308 for the 4-year-old and 41,463 for the 2-year-old.

For each dipole location, we computed the forward field (i.e. the field that would be measured at the MEG sensors in response to a unit current) using a current dipole model in a single-shell volume conductor model (Nolte, 2003), implemented in Fieldtrip (Oostenveld et al., 2011). Forward fields for dipoles oriented in ${\hat{\theta }}_{s}$ and ${\hat{\phi }}_{s}$ were computed independently. Having computed the field magnitude, *b_i_*, at each sensor location/orientation we then calculated the Frobenius norm of the measured field vector as $f_{i}=\sqrt{\overset{N}{\underset{i=1}{\sum }}b_{i}^{2}}$, where *i* indexes MEG channel, *N* is the total number of channels, and *j* indexes the source dipole in the brain. The result is an image showing *f_j_* as a function of location in the brain. Note we use the Frobenius norm as it is the best indicator of the total signal captured across a MEG array (Brookes et al., 2021), and represents an excellent proxy for sensitivity.

These calculations were undertaken for two separate arrays: first we assumed all OPMs measure only the radial component of field (i.e., *N* is simply the total number of OPMs (e.g. 57 for the adult)); second, we assumed that each OPM is triaxial (i.e., *N* is three times the number of OPMs; 171 for the adult). The case for dual-axis arrays is shown in Appendix 2. The computed values, *f_j_*, were normalised by the maximum value to highlight any spatial inhomogeneities in the measured signal, across the brain.

#### 3.1.2. Results

The results of our simulation are shown in Figs. 7c and d. Fig. 7c shows the variation of array sensitivity across the brain for radially-oriented sensors. The left-hand column shows the results for dipoles oriented in ${\hat{\theta }}_{s}$ and the right-hand column shows similar sensitivity maps for dipoles oriented in ${\hat{\phi }}_{s}$. The upper, middle and lower rows show the adult, 4-year-old and 2-year-old, respectively. For an adult, coverage across the brain is approximately uniform, declining with distance from the sensors in areas such as the temporal pole, as expected. This is commensurate with what has been seen experimentally. For example, recent data show that distributed networks can be observed using an OPM array very similar to the one simulated here (Boto et al., 2021); it is unlikely that such networks would be observable if coverage was not spatially uniform. In contrast, for younger individuals, the simulation shows that coverage becomes inhomogeneous, with areas of high sensitivity positioned between the sensors, but areas of lower sensitivity directly beneath the sensors. The spatial signature differs depending on the orientation of the source, as would be expected. This patchy coverage is a direct result of the finite spatial sampling of the array, and the high spatial frequency variation of the magnetic fields measured.

The triaxial array, shown in Fig. 7d, offers more uniform coverage. Whereas a radially-oriented sensor is completely insensitive to a source directly beneath it, a tangential measurement is most sensitive to a source beneath it. So, the areas of low sensitivity introduced in the radial array become “filled” when using a triaxial sensor array. This ensures that the coverage is more uniform. Importantly, dual-axis sensors will have a similar effect, but not to the same extent; specifically, the addition of a tangential axis oriented in ${\hat{\theta }}_{0}$ has little effect on the ${\hat{\theta }}_{s}$ dipoles, but improves coverage of ${\hat{\phi }}_{s}$ dipoles. Likewise, the addition of a ${\hat{\phi }}_{0}$ measurement has little effect on ${\hat{\phi }}_{s}$ dipoles, but improves coverage of dipoles oriented in ${\hat{\theta }}_{s}$. Only the triaxial system offers an improvement for dipoles in both ${\hat{\phi }}_{s}$ and ${\hat{\theta }}_{s}$ (see Appendix 2).

### 3.2. Child measurements

#### 3.2.1. Methods

As a practical demonstration of triaxial sensors, we aimed to measure brain function in a child. A single participant (female, 5 years old) took part in the study, which was approved by the University of Nottingham Medical School Research Ethics Committee.

The paradigm was a parental touch task. On presentation of an auditory cue, the parent (who was sat with the child during the scan) gently stroked the thenar eminence of the subject’s right hand, for approximately 1 s. The inter-trial interval was 5 s and the task was repeated 50 times. Throughout the task, the participant was able to watch their favourite TV show which was presented via a back-projection screen placed approximately 80 cm in front of the subject. The audio cues, and sound for the TV show, were produced using two speakers attached to waveguides in the MSR.

**Fig. 8.**
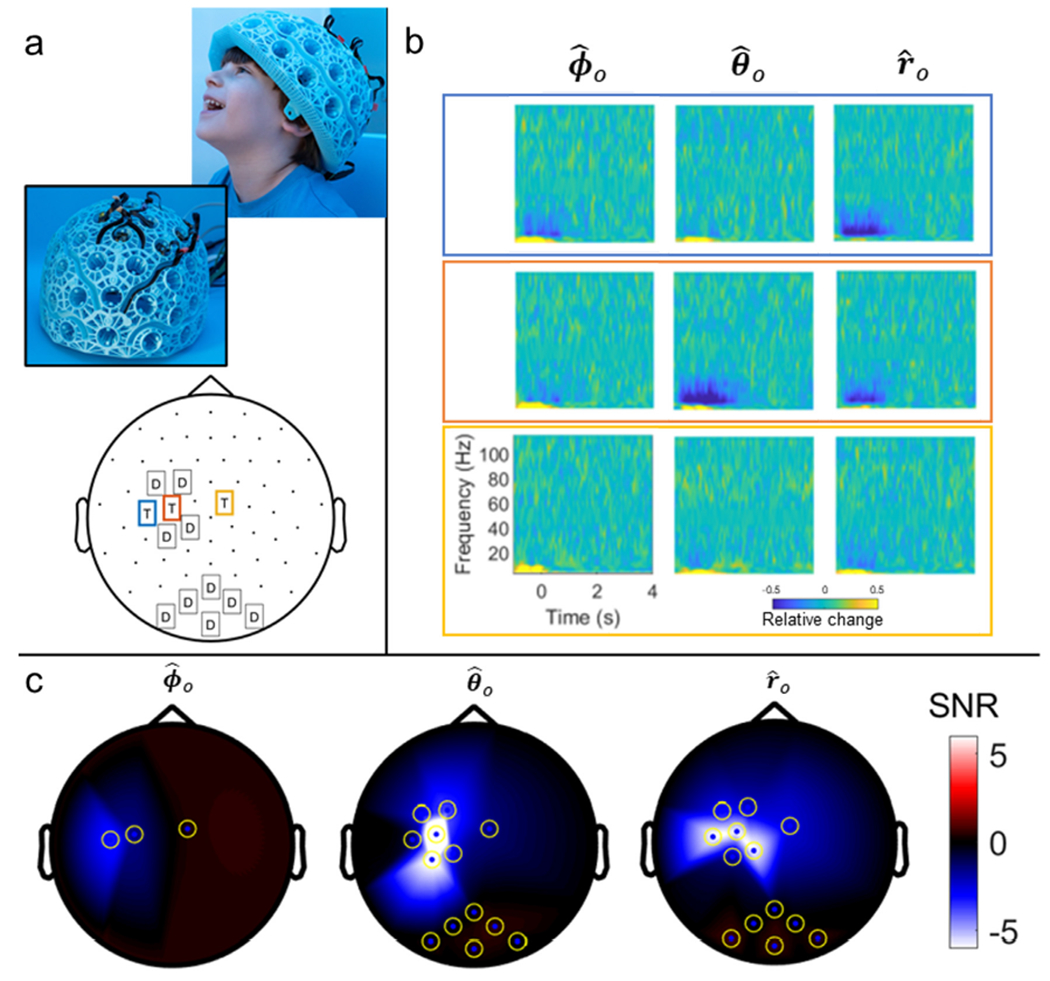
Child measurements: a) Experimental set-up. Top: images showing the helmet (Cerca Magnetics Limited). Inset image shows how sensors were held within the helmet mesh, and the cable channels. Bottom: 2D layout showing locations of dual and triaxial sensors on the helmet. [Note that images of the child are shown with written permission – credit University of Nottingham. This was not the same child that took part in the experiments.] b) TFS from the three triaxial sensors highlighted in blue/orange/yellow in panel a. Channels corresponding to the azimuthal, (left) polar (middle) and radial (right) orientations are shown for each sensor. c) 2D field maps showing SNR values for each channel and all three sensitive axes.

Three triaxial sensors, along with four dual-axis sensors, were mounted above the left sensorimotor cortex. A further six dual-axis sensors were mounted over visual cortex for comparison. Sensors were housed in a ridged helmet (Cerca Magnetics Limited) which had been designed specifically for use in the 4–5-year age group (see Fig. 8a). Specifically, volumetric MRI scans from thirteen 4-year-olds were acquired and used to generate a template brain/head, representative of this age range. The scalp surface was then extracted, expanded radially, and the result used to generate the inner helmet surface (which we estimate would fit 95% of 4–5-year-olds). The helmet itself is formed from a 3D-printed plastic mesh which was designed to minimise weight, allow free-flow of air to the scalp (and the OPMs), and also to protect the OPMs which, once inserted, are enclosed by the mesh. The helmet was also designed with cable-troughs in which the sensor cables run; thus enabling optimised cable management and minimising relative cable movement (which can cause artefact in early implementations of dual-axis OPMs). This design also prevents the child pulling the cables during the scan. The total weight of the helmet (including sensors and cables) is 600 g. The helmet was visually attractive, and the child was happy to wear it.

Having positioned the helmet, data were recorded at a sample rate of 1,200 Hz throughout the paradigm. Prior to data acquisition, the bi-planar coils and reference array were used to null any remnant magnetic field in a volume enclosing the head, to a level below ~1 nT. To gain accurate knowledge of stimulus timing, the parent wore an infra-red visible marker on their thumb, the location of which was tracked throughout the experiment using an optical tracking system (Optitrack).

Note that we had no MRI scan of this participant, and so only sensor-space analysis was undertaken. Data were inspected visually for artefacts; following this, no trials had to be removed. For each channel, a TFS (see part 1 for details) was derived showing the evolution of oscillatory signal amplitude throughout the experiment. For each sensor/orientation a SNR value was estimated: the signal was estimated as the difference in [10–20] Hz oscillatory amplitude, between the −0.5 s < *t* < 0.5 s and 2.5 s < *t* < 3.5 s time windows. The noise was estimated as the standard deviation of the [10–20] Hz amplitude in the 2.5 s < *t* < 3.5 s windows. SNR was calculated for each sensor and sensitive axis independently. All analysis was undertaken using Matlab.

#### 3.2.2. Results

Fig. 8 shows results from our recording in a child. Panel a shows the child’s helmet, alongside a 2D representation of the triaxial (T) and dual-axis (D) sensor locations. Panel b shows raw TFS data from the three triaxial sensors and their three orientations, note the clear power reduction in the [10–15] Hz frequency band during sensory stimulation. Finally, panel c shows SNR values across all sensors, in ${\hat{\phi }}_{0}$, ${\hat{\theta }}_{0}$ and ${\hat{r}}_{0}$ orientations. Note that SNR in the tangential axes is comparable to that in the radial axis, demonstrating clearly the importance of capturing vector fields. This will be addressed further in the discussion below.

## 4. Discussion

OPMs are now well established as an alternative to SQUIDs for the measurement of biomagnetic fields, however the technology remains under development. There is considerable flexibility regarding the design of an OPM and it is not yet clear which variant will ultimately prove to be the best for application in MEG. To date, most OPM-MEG implementations have either used single-axis (equivalent to conventional cryogenic MEG) or dual-axis magnetic field measurements. Here, for the first time, we have demonstrated that a triaxial OPM has considerable promise as the fundamental building block of a MEG system. We have shown that commercial triaxial OPMs can be fabricated with sensitivity approximately equal to that of dual-axis sensors. Our phantom measurements suggest that triaxial OPMs allow the orientation of the local magnetic field vector to be identified with high accuracy. Moreover, our noise floor assessments and in-vivo demonstrations show that the current triaxial implementation has sufficient sensitivity to detect biomagnetic signals from the heart and the brain. In addition, we have shown (in simulation) that a triaxial OPM array offers more uniform coverage than a single or dual-axis sensor, particularly in infants – adding to a growing body of evidence suggesting that triaxial measurements are beneficial. Finally, we have shown (experimentally) the utility of triaxial sensors, in combination with a lightweight ergonomic helmet, for imaging electrophysiological phenomena in a child’s brain.

### 4.1. Assessing the efficacy of triaxial measurement

The finding that triaxial sensors offer sufficient sensitivity to measure biomagnetic signals is an important step. The requirement for three orthogonal field measurements necessitates the use of two laser beams, and the easiest way to achieve this is via a beam splitter. However, this halves the power of the laser beam, and fewer photons leads to less efficient atomic polarisation within the cell. For this reason, we would expect a triaxial sensor to have a higher noise floor compared to single-beam sensors, thus lowering the sensitivity to the magnetic fields of interest. However, our data show that, in practice, the noise floor of the triaxial sensors is comparable to that of the dual-axis sensors (14.9 ± 2.0 fT/sqrt(Hz) compared to 13.8 ± 1.1 fT/sqrt(Hz) in the *z* axis). Results in Figs. 4, 5 and 6 show clearly that the triaxial sensors have sufficient sensitivity to measure cardiac and neuromagnetic fields. In the latter case, Fig. 6 demonstrates that electrophysiological modulations can be delineated even within unaveraged trials. In sum, the results presented demonstrate that the present triaxial implementation has sufficient sensitivity for efficient use as a MEG sensor.

In addition to high sensitivity, our data also show that the triaxial sensor facilitates accurate measurement of the field vector. Our phantom experiments showed that the measured field along all three axes (radial, polar and azimuth) agreed well with theory, with a dipole fitting error of just 5.17 ± 0.04 mm (smaller than the physical size of the dipole itself), and correlation between measured and modelled fields of 0.9879 ± 0.0004 in $\hat{r}$, 0.983 ± 0.001 in $\hat{\theta }$ and 0.965 ± 0.001 in $\hat{\phi }$. The dipole fitting error, and the small discrepancies shown by the correlation values, could result from a number of sources: first, the phantom itself is imperfect: although the dipole wiring employed twisted cables, it is possible that stray field could result from these current carrying wires which would introduce a systematic error in the measured field, and consequently the dipole fit. In addition, there was an expected ~2 mm and ~2 ° error in the placement of OPMs in the phantom slots, which again would give a systematic bias in the measurement. The sequential nature of the measurement (i.e., the repeated use of a small number of sensors, rather than a single simultaneous measurement using many sensors) may also lead to movement between experiments and consequently measurement error. Finally, the finite size of the dipole itself (1 cm) means that it will not behave as a perfect point dipole. Despite these issues, the overall accuracy of the phantom measurement remains encouraging and shows that the OPMs enable an accurate record of field orientation. It should be noted that reducing the phantom data to either dual or single axis and redoing the dipole fit had little effect on the accuracy of the fit. This may seem counter-intuitive but is to be expected since, for a dipole in a spherical conductor, we would not expect the addition of tangential measures to offer any extra information. Rather, the significant advantages of triaxial measurement come from the ability to reduce interference, boost signal, and ensure uniform coverage. Since the phantom measurement was already high SNR, and the dipole positioned so even radial-only measurement could characterise the field it generated, we would not expect a significant difference in accuracy by adding tangential axes.

A significant problem with OPMs, which has not yet been satisfactorily dealt within the literature, is crosstalk between sensors – that is, the disruption of a field measurement at one OPM due to the presence of a second sensor in close proximity. Briefly, OPM measurement relies on a modulation field, which is provided by the on-board sensor coils. The orientation of this modulation field provides the directional sensitivity of the measurement. Critically, all OPMs within an array are fed with a single, coherent, modulation field (to avoid beat frequency artefacts in the measured responses). However, this means that if two sensors (A and B) are close to one another, their modulation fields superimpose. In other words, the field in the cell of sensor A is the summation of the field from the coils of sensor A, and the stray field from sensor B. If the two sensors are sufficiently close, this can result in changes in sensor gain and/or sensitive orientation (see also Eq. 1). In single or dual-axis sensors, once all OPMs in the array are in place (and so crosstalk is occurring), any gain errors resulting from crosstalk can be ‘calibrated out’ by applying a known field in the cell, and artificially correcting the sensor gain to compensate. However, orientation errors cannot be corrected since the sensor cannot offer a complete measurement of the full-field vector. However, in a triaxial sensor, not only can we undertake a three-axis calibration, but we can also correct for orientation errors. Specifically, three separate pulses of magnetic field can be applied using the on-board sensor coils; any signal e.g., generated on the *y*-axis measurement, from an *x*-axis pulse, can then be characterised, and corrected in software. This orthogonalisation step – implemented in the present sensors – means that (if the on-board coils generate truly orthogonal fields) the sensor will provide a true estimation of not only field magnitude, but also field direction. This is a significant advantage of triaxial sensors over single or dual-axis OPMs since both gain and orientation errors, generated by crosstalk, can be corrected fully. In the data shown, the high correlation between the data and model in our phantom suggest that crosstalk errors have been adequately dealt with by this procedure.

One important point relates to data modelling, and accounting for the multiple beams within the sensor. To minimise interaction, the 2 laser beams are spatially displaced by 0.65 mm (see Fig. 1). However, this means that, effectively, the *x* and *z* components of field are measured at slightly different locations whilst the *y* component is measured simultaneously at 2 locations, and the results averaged. Here, for data modelling, we have assumed that this 0.65 mm spatial error is small compared to other errors in sensor localisation; indeed this is likely given the accuracy with which we could position sensors. However, with 3D-printed helmets offering sub-mm localisation accuracy, in future studies accounting for this spatial discrepancy in forward field modelling may become important. Simulations (see Appendix 1) suggest that, for dipoles at an average cortical depth, this spatial discrepancy generates around a 2 % error in the forward field, compared to ~3 % for shallow sources. These errors could be easily accounted for, by building the spatial difference in field location into the forward field model. This should be considered for future studies, particularly if this becomes the dominant source of spatial error.

In summary, the high sensitivity to magnetic field, accurate vector measurement, and insensitivity to crosstalk make triaxial sensors a very attractive solution for MEG measurement. Their small and lightweight packaging ensures that the well-known advantages of OPMs are maintained. Further, the triaxial sensors can be fabricated with relatively little extra cost compared to dual- or single-axis OPMs (a triaxial sensor still only requires a single cell, laser and coil set etc., and additions such as an additional photodiode and beam splitter are relatively inexpensive). Thus, it is likely that triaxial sensors could become the hardware of choice for MEG applications.

### 4.2. Towards implementation of a paediatric OPM-MEG system

The ability to make high fidelity MEG measurements in infants is, arguably, the biggest advantage of OPM-MEG over conventional scanners. Adaptability to different head sizes, coupled with motion robustness, offers high sensitivity, improved spatial resolution, and a system that children can tolerate. However, to date relatively few paediatric OPM studies have been performed (Hill et al, 2019) and the design of a paediatric OPM array is far from complete.

Here, our simulations showed that the proximity of sensors to the brain in an infant head can lead to significant sampling problems. The idea that closer sensors are problematic is counter-intuitive in MEG, since the closer a sensor gets to the brain, the larger the measurable magnetic field, and the more focal its spatial pattern. Thus, we get better SNR and spatial resolution. However, our simulations show that in an infant head, where distance from the sensor to the brain can be ~5 mm, the spatial patterns become too focal. Any pragmatic OPM-MEG system involves a finite number of sensors (currently around 50 (Tierney et al., 2020)) and there will always be gaps between sensors. Thus, these highly focal fields become poorly sampled. As shown in Fig. 7, the result is that areas directly under a radially-oriented OPM begin to exhibit poor sensitivity compared to the brain regions between sensors. Consequently, the sensitivity profile varies across the cortex. This is not an issue in conventional MEG, because sensors are further away to allow for a thermally-insulating gap between the scalp and sensors; it is less of an issue for OPM-MEG in adults because the brain is around 15 mm beneath the skull surface (see Fig. 7a), and it is not a problem in EEG since the electric potentials are spatially smeared by the presence of the skull. However, for paediatric OPM-MEG, where the brain is very close to the scalp, our simulations suggest a significant caveat, with a strong likelihood that electrical phenomena in the brain could be missed if the region of interest falls within an area of low sensitivity. However, not only do triaxial measurements increase the overall amount of signal captured (by virtue of the fact that we have three times more field measurements) but they also provide more uniform coverage. Obviously, one could equally achieve more uniform coverage by either adding more radial sensors, or stepping sensors off the head; however, the former would likely be impractical since one might need many hundreds of sensors (significantly increasing cost), whereas the latter would sacrifice sensitivity by moving sensors away from the brain. For these reasons, the addition of a triaxial measurement would appear to be the most attractive, and cost-effective solution to ensure even coverage.

Previous work (Brookes et al., 2021; Nurminen et al., 2013) has suggested that triaxial measurement offers improved differentiation between fields originating in the brain, and fields generated by sources of external interference. Specifically, whilst tangential field measurements offer little additional information (beyond the radial field) for sources in the brain (apart from the sampling problems described above), they do provide more information on sources of interference. This means that, e.g. artefacts from environmental sources (e.g., laboratory equipment) simulus equipment (e.g, a median-nerve stimulator) or even biological sources of no interest (e.g., the field from the heart) can be better distinguished from genuine neural souces, if triaxial measurements are made. This extra information can then be exploited by interference rejection techniques such as signal-source separation (Nurminen et al., 2013) or source-localisation algorithms (Brookes et al., 2021) and this enables a better SNR.

In OPM-MEG, an additional source of artefact comes from movement (Rea et al., 2021; Seymour et al., 2021). If an OPM rotates in a static magnetic field, or moves in a field gradient, it will measure a changing field. This unwanted signal is supressed by the use of magnetic field nulling which attempts to reduce the field to zero and thus nullify the problem. However, field nulling is never perfect and small remnant fields often remain inside a MSR, which generate artefacts as an individual moves. In practice, these movement artefacts manifest in frequency bands that overlap with movement; i.e. they rarely affect high frequencies (e.g. alpha and upwards) but can affect theta and delta signals. However, when imaging children we note that 1) the frequency of movement may differ from that of adults, 2) the frequency of neural oscillations tends to be somewhat lower (therefore overlapping with movement artefacts) and 3) children tend to move more. We might then expect that movement artefacts will be worse in infants than we have typically seen in adults, and may obfuscate signals of interest. Here again, triaxial measurement may offer advantages: the movement artefact manifests as a moving external magnetic field and, just as for sources of external interference, these artefacts are better differentiated from true brain activity if triaxial measurements are undertaken. For this reason, triaxial measurement is likely to offer a better route to eliminating movement artefact, which may provide a further advantage for scanning infants.

## 5. Conclusion

In conclusion, we have demonstrated the feasibility of a commercially-available triaxial OPM to make measurements of the vector magnetic field from the human brain. Our results confirm both high accuracy and sensitivity. Further, we have demonstrated (in simulation) how triaxial measurement is likely to offer improved cortical coverage, especially in infants and children, and we have introduced an ergonomic child-friendly OPM-MEG helmet design which allowed triaxial measurement in a five-year-old. Overall, the data demonstrate that this type of sensor is a significant improvement over the previously available dual-axis variants. It offers benefits for the design of OPM arrays for paediatric applications and constitutes an excellent building block for the design of future MEG imaging platforms.

## Supplementary Material

1

## Data Availability

All data and code available on request to the authors.

## Abbreviations:

OPM: optically-pumped magnetometer
MEG: magnetoencephalography
