# Supplementary material for: Triaxial detection of the neuromagnetic field using optically-pumped magnetometry: feasibility and application in children
Source: Neuroimage. Author manuscript; Available in PMC 2022 May 26. (PMC9135302; doi:10.1016/j.neuroimage.2022.119027)
Supplement: 1 [file NIHMS1805131-supplement-1.pdf]

Appendix 1: Spatial error and the effect on lead fields

The triaxial sensor relies on two orthogonal laser beams, which probe the atomic vapour, and consequently the magnetic field, at two locations within the cell. The two beams are offset by 0.65 ± 0.25 mm in a tangential orientation (e.g. $\widehat{\boldsymbol{\theta}_s}$, $\widehat{\boldsymbol{\phi}_s}$, or a combination of the two). This means that, in practice, there is a spatial discrepancy (maximum 0.65 mm) between the assumed location at which the field is measured, and the real location. Here, these discrepancies are not accounted for in data modelling. The aim of this appendix is to estimate an upper limit on the error that this spatial discrepancy is likely to introduce to the measured field.

We undertook a simulation (in Matlab): Current dipoles were simulated at ~30,000 points beneath the brain surface. For each dipole, a forward field was calculated at 59 triaxial sensors located on the scalp. This calculation was then repeated with the sensors shifted in location by 0.65 mm, in either $\widehat{\boldsymbol{\theta}_o}$ or $\widehat{\boldsymbol{\phi}_o}$. For each dipole, this resulted in two sets of forward fields; the difference was computed, and its Frobenius norm (across channels) calculated. The ratio of the norm of the error, to the norm of the original forward field was then derived as an approximation of the fractional error caused by the spatial discrepancy. These errors were estimated for all dipole locations, with dipoles oriented in both $\widehat{\boldsymbol{\theta}_s}$ and $\widehat{\boldsymbol{\phi}_s}$. We probed dipoles on two surfaces, a “shallow” surface ~5 mm beneath surface of the brain, and a deeper surface at ~2 cm depth.

Results are shown in Fig. A1. The figure is split into four panels: the upper panels show forward field error for shallow sources; the lower panels show equivalent results for deeper sources. The left-hand panels show the case when the sensor location error is in $\widehat{\boldsymbol{\theta}_o}$; the right-hand panels show sensor location error in $\widehat{\boldsymbol{\phi}_o}$. In all cases, the line plots show a histogram of the errors recorded over all dipole locations. The maps show the spatial signature of that error over the brain (all maps scaled between 0 and 5%). As expected, the sensor location error has the greatest effect for shallow sources, but is less than 5% for all dipoles. For deeper sources the error was reduced with all dipoles having less than 2.5% forward field error. This difference results because the magnetic fields generated by a deep dipole are spatially diffused, and so a location error has proportionally less effect. Also, as would be expected, a sensor location error in the $\widehat{\boldsymbol{\theta}_o}$ direction has the largest effect on a dipole oriented in $\widehat{\boldsymbol{\phi}_s}$. Likewise, a sensor location error in the $\widehat{\boldsymbol{\phi}_o}$ direction has the largest effect on a dipole oriented in $\widehat{\boldsymbol{\theta}_s}$. This is a result of the spatial signature of the fields generated.

In conclusion, errors on the forward field caused by uncertainty in the location at which field is probed, are relatively small. Nevertheless, errors of around 5% may have an effect on source modelling, particularly when using adaptive solutions. For the purposes of this paper, with only 4 triaxial sensors, source localisation was fundamentally limited and so it is unlikely that these subtle spatial dominate. However, if a large array of triaxial OPMs is constructed, these errors may be significant, and could prevent realisation of the precision of OPM-MEG – particularly for shallow sources. For this reason, we would advise that the spatial discrepancy of the two beams is taken into account in future source models of triaxial MEG data.

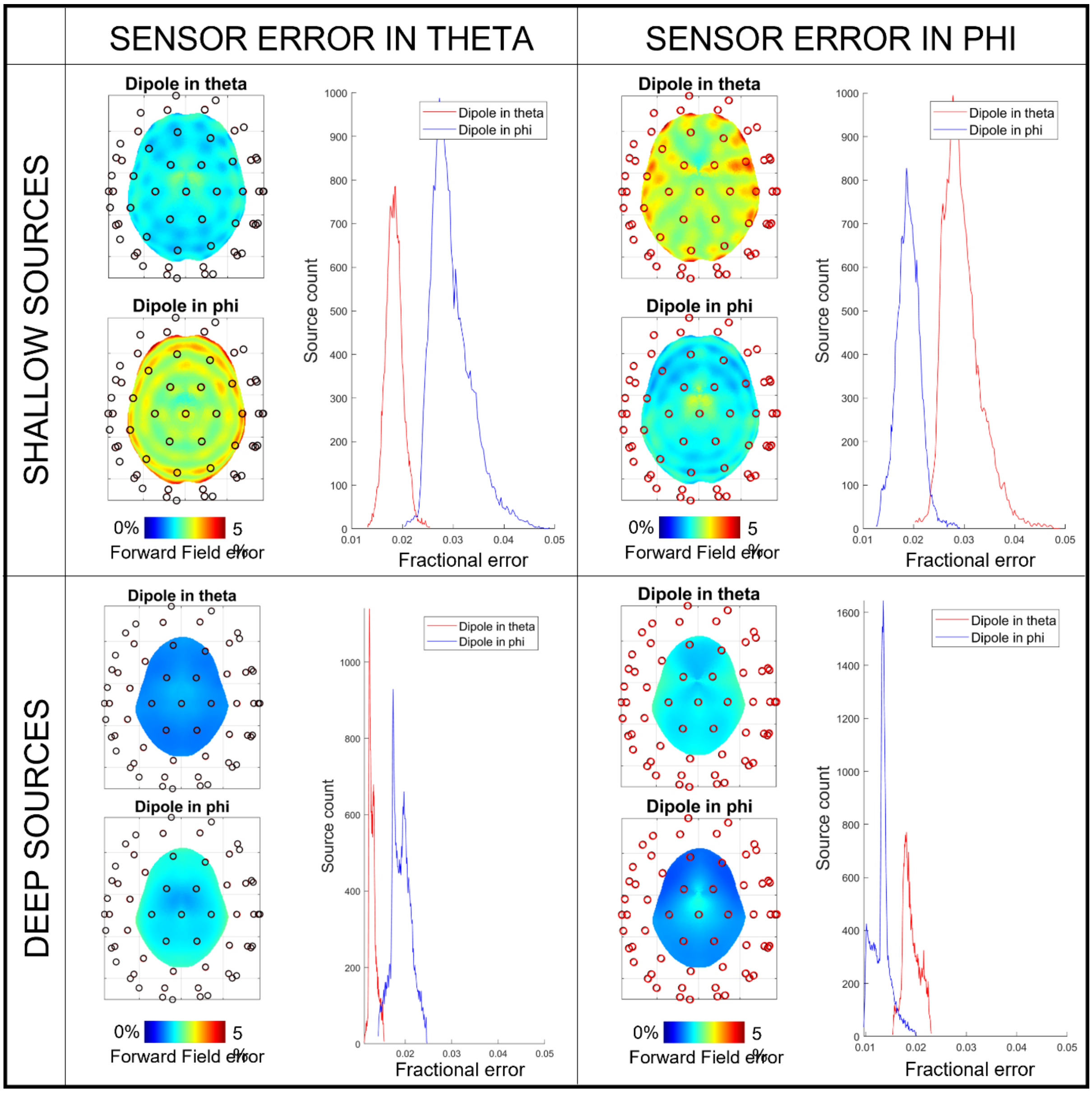

Fig. A1: Forward field error generated by uncertainty on source position. *Upper plots show dipoles on a shallow surface in the brain; lower plots show dipoles on a deeper surface. Left plots show sensor positions shifted in the* $\widehat{\boldsymbol{\theta}_o}$ *direction. Right plots show sensor uncertainty in* $\widehat{\boldsymbol{\phi}_o}$*. For each of the four quadrants, the brain plots show the spatial distribution of the forward field error across the dipolar surface. The case for dipoles in* $\widehat{\boldsymbol{\theta}_s}$ *is shown at the top;* $\widehat{\boldsymbol{\phi}_s}$ *at the bottom. The line plots show histograms of forward field error across all dipoles in the simulation. Note errors are smaller for deeper sources but remain relatively small (<5%) in all cases.*

Appendix 2: Dual axis infant simulation results

Results in Fig. 7 compare radially oriented sensors with triaxial sensors, showing that the latter offer better coverage. However, most OPMs offer dual axis measurement, and it proves instructive to add this additional comparison to the simulation. On average when measuring MEG signals, the radially oriented magnetic field is larger than either of the two tangential components. Thus, it makes sense to always measure this radial component. However, this leaves the open question – when using dual axis sensors, which of the two tangential components ($\widehat{\boldsymbol{\theta}_o}$ or $\widehat{\boldsymbol{\phi}_o}$) should be measured? (Since we cannot

measure both.) Here, we simulated two dual axis OPM-MEG systems, the first in which we measure the radial and polar orientations of field; in the second we measure the radial and azimuth. Aside from the sensitive orientations, the simulation was identical to that in section 2.2. The simulation was applied to the four-year-old template brain only.

Results are shown in Fig. A2. The images show the norm of the forward field, scaled by its maximum, for dipoles across the brain. The left-hand images show the case for dipoles oriented in $\widehat{\boldsymbol{\theta}_s}$; the right-hand images show dipoles oriented in $\widehat{\boldsymbol{\phi}_s}$. The upper panel shows a radial only system (result repeated from Fig. 7). Note again the patchy coverage of the brain for both sets of dipoles. The upper centre panel shows a system where fields are measured along the radial and polar ($\widehat{\boldsymbol{\theta}_o}$) axes. Note that the addition of this tangential axis has little effect on the $\widehat{\boldsymbol{\theta}_s}$ dipoles, but improves coverage for dipoles oriented in $\widehat{\boldsymbol{\phi}_s}$. The lower centre panel shows a system where fields are measured along the radial and azimuthal ($\widehat{\boldsymbol{\phi}_s}$) axes. Here, we see little effect on $\widehat{\boldsymbol{\phi}_s}$ dipoles, but improved coverage for dipoles in $\widehat{\boldsymbol{\theta}_s}$. Only the triaxial system offers an improvement for dipoles in both $\widehat{\boldsymbol{\phi}_s}$ and $\widehat{\boldsymbol{\theta}_s}$. In summary, dual axis systems ameliorate the coverage problem, only if dipole orientation and sensor orientation are matched. In practice this is impossible, and so dual axis sensors will always leave gaps in coverage in younger subjects. Triaxial sensors, alternatively, offer better homogeneity.

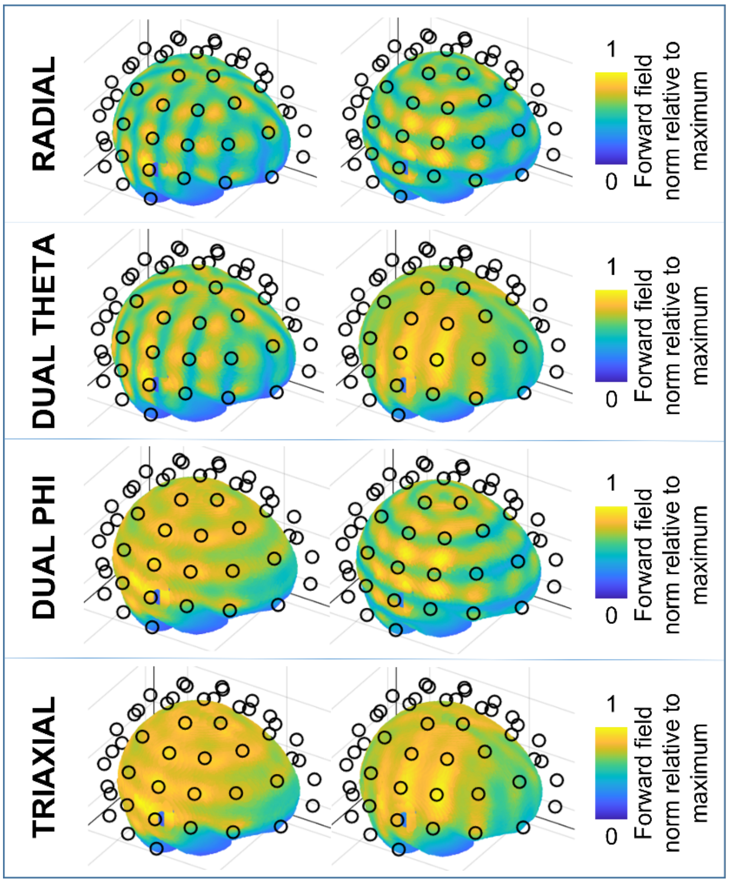

Fig. A2: Sensor coverage for a 4-year-old, using dual axis OPM-MEG. *The upper (Radial) and lower (Triaxial) panels repeat results shown in Fig. 7. The central 2 panels show equivalent results for two hypothetical dual axis OPM-MEG systems. The upper centre panel shows a system which measures radial and polar field; the lower centre panel shows a system which measures radial and azimuthal field. Left hand images show dipoles oriented in* $\widehat{\boldsymbol{\theta}_s}$*; right hand images show dipoles oriented in* $\widehat{\boldsymbol{\phi}_s}$*.*
